# Adhesion of *Trypanosoma cruzi* Trypomastigotes to Fibronectin or Laminin Modifies Tubulin and Paraflagellar Rod Protein Phosphorylation

**DOI:** 10.1371/journal.pone.0046767

**Published:** 2012-10-04

**Authors:** Eliciane C. Mattos, Robert I. Schumacher, Walter Colli, Maria Julia M. Alves

**Affiliations:** Departamento de Bioquímica, Instituto de Química, Universidade de São Paulo, São Paulo, Brazil; Faculdade de Medicina, Universidade de São Paulo, Brazil

## Abstract

**Background:**

The unicellular parasite *Trypanosoma cruzi* is the causative agent of Chagaś disease in humans. Adherence of the infective stage to elements of the extracellular matrix (ECM), as laminin and fibronectin, is an essential step in host cell invasion. Although members of the gp85/TS, as Tc85, were identified as laminin and fibronectin ligands, the signaling events triggered on the parasite upon binding to these molecules are largely unexplored.

**Methodology/Principal Findings:**

Viable infective parasites were incubated with laminin, fibronectin or bovine serum albumin for different periods of time and the proteins were separated by bidimensional gels. The phosphoproteins were envisaged by specific staining and the spots showing phosphorylation levels significantly different from the control were excised and identified by MS/MS. The results of interest were confirmed by immunoblotting or immunoprecipitation and the localization of proteins in the parasite was determined by immunofluorescence. Using a host cell-free system, our data indicate that the phosphorylation contents of *T. cruzi* proteins encompassing different cellular functions are modified upon incubation of the parasite with fibronectin or laminin.

**Conclusions/Significance:**

Herein it is shown, for the first time, that paraflagellar rod proteins and α-tubulin, major structural elements of the parasite cytoskeleton, are predominantly dephosphorylated during the process, probably involving the ERK1/2 pathway. It is well established that *T. cruzi* binds to ECM elements during the cell infection process. The fact that laminin and fibronectin induce predominantly dephosphorylation of the main cytoskeletal proteins of the parasite suggests a possible correlation between cytoskeletal modifications and the ability of the parasite to internalize into host cells.

## Introduction

The identification of molecules involved in the multiple interactions of *Trypanosoma cruzi* with host cells, as well as their resulting signaling pathways, is fundamental to the understanding of the mammalian infection by the parasite. *T. cruzi*, the causative agent of Chagas` disease, is transmitted to humans by blood sucking triatomines (insect vector), ingestion of contaminated food or blood transfusion. The parasite multiplies as epimastigotes in the midgut of the insect digestive tract and differentiates to metacyclic trypomastigotes in the hindgut, being eliminated with the feces and urine after a blood meal. The excreted parasites can infect mammalian host cells through the bite wound or the mucous membranes. Inside cells, the parasites multiply as amastigotes and differentiate to trypomastigotes. The parasites are released in the circulation upon rupture of the host cells and may invade new cells or be ingested by the insect vector where they differentiate into epimastigotes [1]. Thus, during this complex life cycle, the parasite is exposed to different environments and has to respond adequately in order to survive. Among the possible responses, post-translational modification of proteins appears to be operative.

Post-translational modifications of proteins provide an efficient tool to regulate the activity of key proteins, with reversible phosphorylation being a frequent one. The large amount of kinases (190 protein kinase genes) and phosphatases (86 protein phosphatase genes) in the *T. cruzi* genome suggests a major role of the protein phosphorylation cycle in signaling events [2], [3]. Accordingly, from the 119 proteins with sequenced phosphorylation sites described in *T. cruzi* epimastigotes, 6 are protein kinases or phosphatases [4]. Analysis of the phosphoproteome during differentiation of epimastigotes to metacyclic trypomastigotes, revealed 84.1% of the phosphorylated sites in serine residues, the others being threonine (14.9%) and tyrosine (1%) [5]. In general, although percentages may vary, serine and threonine are the preferential modified residues, as detected for example in the analysis of 491 phosphoproteins from bloodstream forms of *T. brucei* [6] or 445 putative distinct phosphoproteins from *Leishmania* [7].

Despite the importance of protein post-translational modifications, much less is known about their role in *T. cruzi* signaling during parasite invasion. A decrease in the amount of trypomastigote-induced host tyrosine phosphorylated proteins (∼234, 205 and 50 kDa) was described [8] and treatment with inhibitors of protein tyrosine phosphatases impairs infection by *T. cruzi* [9]. Specific PT1b inhibitor (BZ3) and anti-sense oligonucleotides of calcineurin B pointed out to the role of PT1b and PT2A [10] in trypomastigote invasion. On the other hand, the involvement of kinases in the process was suggested by the phosphorylation of a non identified 175 kDa peptide during invasion of metacyclic trypomastigotes, the infective stage present in the invertebrate digestive tract [11].

Adhesion of *T. cruzi* trypomastigotes to elements of the extracellular matrix (ECM), such as fibronectin [12], laminin-1 [13], heparan sulfate [14], galectin-3 as enhancer of the laminin-binding [15] and collagen IV [16] is well established and appears to be important for parasite infectivity. Also, it has to be stressed that the ability of *T. cruzi* to survive in the mammalian host is partially due to its capacity to invade host cells and many molecules have been implicated in this step. Particularly, some belong to the subgroup II of the Gp85/Trans-sialidase superfamily, encoded by a high polymorphic supergene family composed of approximately 700 genes and equal number of pseudogenes [17]. Members of subgroup II have no trans-sialidase activity, but are able to bind to cytokeratins [18], collagen, fibronectin and laminin-1, and encompass the Tc85 family of glycoproteins involved in *T. cruzi* invasion [19]. Advantage of this information has been taken to identify proteins that are differentially phosphorylated during adhesion of trypomastigotes to fibronectin and laminin in order to understand better the invasion process.

The importance of the cytoskeleton in signal transduction is increasingly being recognized. In trypanosomatids there is a characteristic sub-pellicular corset of microtubules linked to each other and to the plasma membrane, formed by α- and β-tubulin subunits. The flagellum contains a second microtubule organization composed by the conserved structure of “9+2″ microtubules (axoneme). This structure together with the paraflagellar rod, which runs alongside the axoneme, is linked to the cell body by other cytoskeletal elements [20]–[23]. It has been recently proposed that in addition to an important structural function, the cytoskeleton may have a role in mediation of signaling transmission [23].

Thus, a thorough analysis of phosphorylation/dephosphorylation events in *T. cruzi* trypomastigote cytoskeletal major proteins, as α- and β- tubulins and paraflagellar rod proteins, has been undertaken for the first time. Moreover, involvement of the ERK pathway is suggested.

## Materials and Methods

### Ethics Statement

This study was carried out in strict accordance with the recommendations in the Guide for the Care and Use of Laboratory Animals of the National Institutes of Health (NIH Publication 8523). The protocol was approved by the Animal Ethics Committee of the Instituto de Química, Universidade de São Paulo (Permit Number: 17/2011), which follows regulations issued by CONCEA (Conselho Nacional de Cuidado e Experimentação Animal), Brazil.

### Reagents

Laminin-1 from Engelbreth-Holm-Swarm (EHS), Protease Inhibitor Cocktail, anti-mouse-IgG antibodies conjugated to Peroxidase, anti-phospho-Serine (pS) and anti- α-tubulin antibodies were purchased from SIGMA-ALDRICH; human plasma fibronectin from Gibco; DeStreak rehydration solution from GE Healthcare Life Science; PRO-Q Diamond Phosphoprotein Gel Stain, anti-phospho-Threonine (pT) and anti-phospho-Tyrosine (pY) antibodies from Invitrogen; and anti-phospho ERK antibody from Cell Signaling.

### Cell Cultures and Incubation with Fibronectin and Laminin-1

*Trypanosoma cruzi* Y strain was maintained by infection of LLC-MK_2_ cells [24] in DMEM with 2% of fetal bovine serum (FBS). Five days after infection, trypomastigotes released into the medium were collected for further incubation. Tissue culture plates (8 wells plates) were coated with 2.5 µg of Bovine serum albumin (BSA), human plasma fibronectin or laminin-1 in 0.2 M NaHCO_3_, pH 9.5 for 14 h at 4°C. After washing three times with PBS, 1×10^9^ parasites in 1 ml of DMEM supplemented with 2% FBS were added to each well and incubated at 37°C in a 5% CO_2_ atmosphere. After the desired time, the parasites were collected and washed with PBS containing phosphatase (5 mM NaF, 2 mM Na_3_VO_4_ and 50 µM Na β-Glycerophosphate) and protease inhibitors (Protease Inhibitor Cocktail plus 1 mM PMSF). The parasite pellet was kept at −80°C. Three independent experiments were performed for each treatment. The triplicates have been compared with each other by the Image Master Platinum 7 software.

### Protein Extraction and Two Dimensional Electrophoresis (2D-PAGE)

DeStreak rehydration solution, containing phosphatase and protease inhibitors as described, was added to the frozen parasite pellet. Parasites were submitted to ultra sonic disruption for 15 min on ice followed by centrifugation at 20,000×*g* for 7 min at 4°C. The supernatant was collected and proteins were quantified by Bradford [25]. To ensure the quality of the extracts, they were first analyzed by 10% SDS-PAGE [26].

The first dimension (IEF) was performed using Ettan IPGphor 3 System (GE Healthcare). 13 cm DryStrip (pH 4–7) immobiline strips were loaded with 450 µg of proteins from *T. cruzi.* IEF was carried out by ramping 1000V for over 800Vh; ramping 8000V for over 11300Vh; holding at 8000V for 5400Vh; yielding a total of about 35000Vh, as suggested by the GE Healthcare instructions.

For the second dimension, IPG strips were previously incubated with 1% DTT in the equilibrium buffer (75 mM Tris-HCl pH 8.8; 6 M urea; 29% glycerol and 2% SDS) for 15 min, followed by 15 min in equilibrium buffer supplemented with 2.5% iodoacetamide. The strips were washed with water and transferred to the top of 12% SDS-Polyacrylamde gel, overlaid with 0.5% agarose solution and submitted to electrophoresis. After running, the gels were stained with PRO-Q Diamond Phosphoprotein Gel Stain to identify phosphoproteins and then colloidal Coomassie was added for total protein staining. Gels were scanned with Typhoon Trio (GE Healthcare) and ImageScanner III (GE Healthcare) to identify phosphoproteins and total proteins, respectively. Gel analyses were carried out in ImageMaster 2D Platinum 7.0 (GE Healthcare). A standardized spot with 5% statistical significance test in ANOVA (analysis of variance) was considered to determine significant changes in phosphorylation levels. Two experiments treating *T. cruzi* extracts with alkaline phosphatases (Thermo Scientific and Roche) eliminated most of the Pro Q recognized phosphorylated spots.

### Protein Identification by LC-ESI-Q-TOF

The spots of interest were manually excised from the gel and treated as follows: (1) Destaining of the spots with 50% methanol-2.5% acetic acid; (2) Peptide reduction in 10 mM dithiothreitol (DTT) and peptide alkylation with 50 mM iodoacetamide; (3) Polypeptide enzymatic hydrolysis with 0.4 or 0.8 µg trypsin (PROMEGA), accordingly to the spot size, and (4) extraction of the peptides from the gel spots. For peptide sequencing, the mass spectrometer ESI-Quad-TOF coupled to UPLC system was used at Mass Spectrometry Laboratory of LNBio, CNPEM/ABTLuS (Campinas, Brazil). The mass spectra were processed through the program ProteinLynx V 2.1 (Waters) and analyzed with the MASCOT MS/MS Ion Search (http://www.matrixscience.com). The parameters used were: enzyme, trypsin; allow up 1 missed cleavage; fixed modification, carbamidomethyl (C); variable modifications, oxidation (M), phosphorylation (S/T) and phosphorylation (Y); peptide tolerance, ±0.1Da; MS/MS; tolerance: ±0.1Da; and peptide charge, 1+2+ and 3+. The database Tcruzi2011, included in Tritryp database, was used to identify the proteins.

### Immunoprecipitation, Immunoblotting and Immunofluorescence Techniques

Extracts of 1x10^9^ trypomastigotes previously incubated with fibronectin, laminin or BSA, as described above, were ressuspended in 500 µL of Lysis Buffer (50 mM Tris-HCl pH 8.0; 150 mM NaCl and 1% CHAPS, supplemented with Protease/Phosphatase Inhibitor cocktail and submitted to ultra sonic disruption for 15 min on ice, followed by centrifugation at 20,000×*g* for 7 min at 4°C. To reduce unspecific binding, the samples were first incubated with 50 µL of Protein A-Sepharose 4 fast flow resin (GE Healthcare Life Science) for 30 min at 4°C, followed by centrifugation to remove the resin. The supernatants were incubated with 5 µL of anti-α-tubulin antibodies or 5 µL of anti-paraflagellar-rod protein monoclonal antibody (anti-PAR Mab) and 50 µL of Protein A-Sepharose for 14 h at 4°C. After extensive wash with Lysis Buffer, the resin was ressuspended in 50 µL of Laemmli Buffer (60 mM Tris-HCl pH 6.8; 2% SDS; 10% Glycerol; 5% β-mercaptoethanol; 0.01% Bromophenol Blue) followed by SDS-PAGE and immunoblotting with a mixture of anti-pS, -pT, -pY, anti-α-tubulin antibodies or anti-PAR Mab. To raise Anti-PAR Mab, Balb/c mice were inoculated every other week with 50 µg of a trypomastigote extract, during 4 weeks. BALB/c female mice were housed under barrier conditions at the animal care facility at the Instituto de Química, Universidade de São Paulo (IQUSP, Brazil). The hybridomes were obtained accordingly to the literature [27]. The specificity of the antibody was verified by immunoprecipitation of trypomastigotes extract with the antibody, followed by MS/MS sequencing of the 3 bands separated by SDS-PAGE.

The presence of phosphorylated ERK was detected in immunoblots of trypomastigote extracts as described above developed with anti-phospho ERK and anti-ERK antibodies. To analyze soluble and polymerized molecules, pellets of frozen parasites were ressuspended in homogenization buffer (20 mM Tris-HCl pH 7.5, 2 mM EDTA, 10 mM EGTA, 0.25 M sucrose, Protease/Phosphate Inhibitor Cocktail, followed by ultracentrifugation at 110,000*×g* for 40 min at 4°C. The supernatant (S) and the pellet (INS) containing soluble and polymerized tubulins and PAR, respectively, were separated and the pellet ressuspended in RIPA buffer (50 mM Tris-HCl pH 7.5, 150 mM NaCl, 0.1% SDS, 0.5% Sodium Deoxycholate, 1% Trition X-100 plus the Protease/Phosphatase Inhibitors Cocktail), as described in reference [28]. Both fractions were immunoprecipitated with 5 µL α-tubulin antibodies or 5 µL anti-PAR Mab and submitted to SDS-PAGE, followed by immunoblotting with a mixture of anti-phosphorylated-amino acids, anti-α-tubulin or anti-PAR antibodies. The reactivity was developed with specific peroxidase conjugated anti-mouse-IgG antibodies. The band intensity was quantified by Image J program. The ratio showed in the figures was calculated by the relative intensity of phosphorylated protein (α-tubulin, PAR or ERK1/2) in each treatment (fibronectin or laminin) divided by the relative intensity of phosphorylated protein (α-tubulin, PAR or ERK1/2) in the control (BSA). The relative intensity of phosphorylated protein is the ratio between intensity of phosphorylated proteins (using antibodies pS, pT and pY) and the intensity in each lane of a given specific band related to the amount of protein: for ERK experiments, GAPDH was used; for α-tubulin experiments, the IgG heavy chain sign was employed, and for PAR experiments, the intensity of total PAR was used. Densitometric analysis of protein bands was performed using ImageJ.

For the immunofluorescence, parasites were incubated with fibronectin-, laminin- or BSA-coated coverslips. The non adhered parasites were removed by washing with PBS and fixed with 2% p-formaldehyde for 15 min. After extensive washing, the cells were permeabilized with TBS-0.1% Triton X-100, blocked with 1% BSA and incubated with a mixture of anti-pS, pT and pY, anti α-tubulin or anti-PAR antibodies. The reactivity was developed with specific anti-IgG antibodies conjugated to Alexa-Fluor 555 (Molecular Probes) and the nucleus stained with 20 µg/mL of 4',6-diamidino-2-phenylindole, dilactate (DAPI-Invitrogen). Images taken in a Nikon Eclipse E600 microscope were obtained under identical settings and were submitted to deconvolution with Huygens Essential image processing program. The number of modified parasites upon incubation with fibronectin and laminin was quantitated in 6 independent microscopic fields covering approximately 240 parasites.

The immunoblotting and the immunofluorescence assays have been analyzed by the Student's t-test.

## Results

### Adhesion of *T. cruzi* Trypomastigotes to Fibronectin or Laminin-1 Leads to Changes in the Phosphorylation Status of Proteins

**Figure 1:**
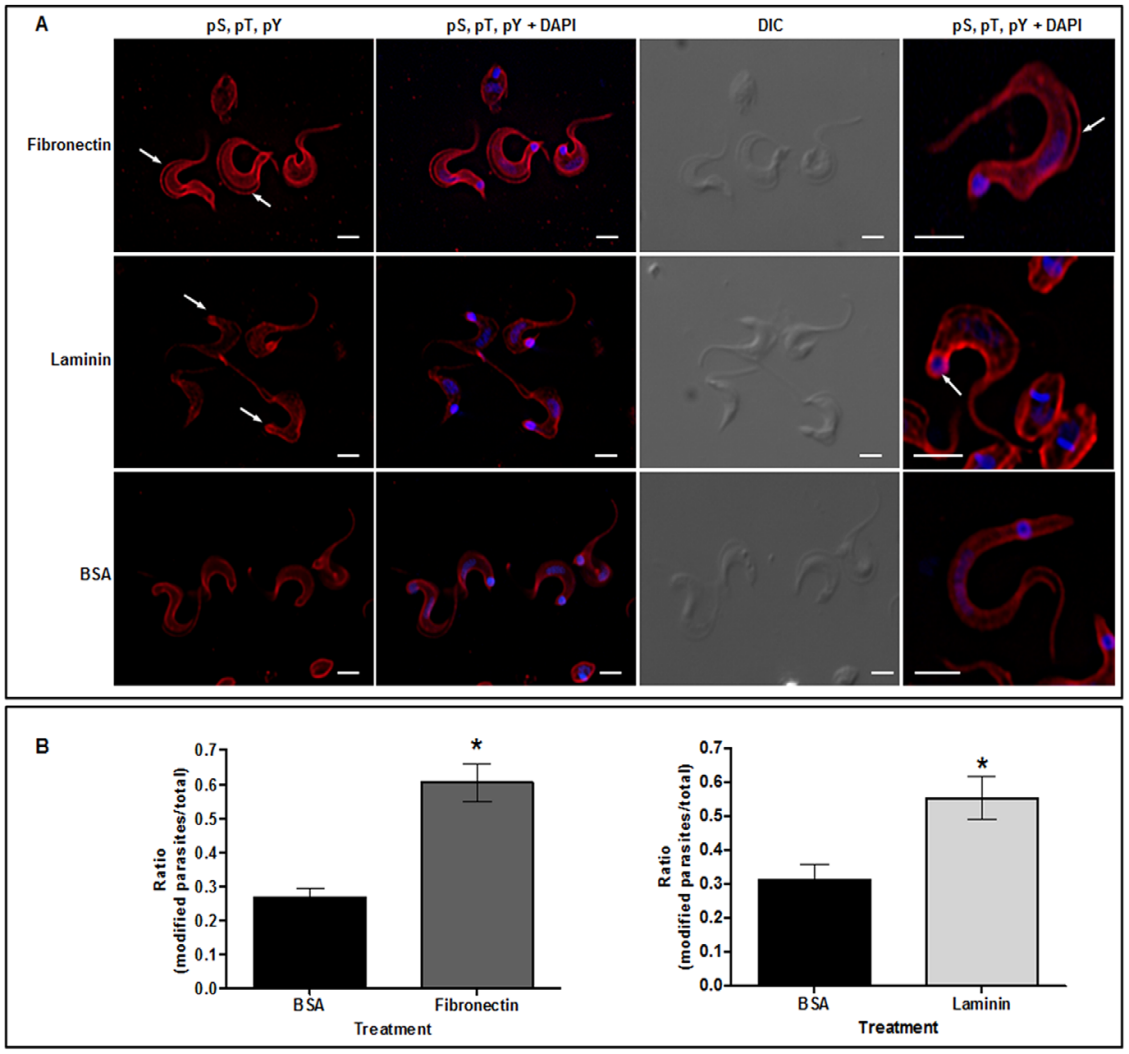
Phenotype of *T. cruzi* trypomastigotes incubated with fibronectin, laminin or BSA. Incubation was for 2 h: (**A**) Localization of phosphoproteins is shown by the reactivity with anti-pS, -pT, -pY antibodies (red); nucleus and kinetoplast stained with DAPI (blue) and DIC images were also shown; white bars represent 3.2 µm. (**B**) Quantitation of parasite modifications (arrows) due to treatment with fibronectin and laminin, respectively, as compared to BSA treatment is shown; 6 fields with approximately 40 parasites each have been examined; asterisks represent a p<0.001 when experimental points were compared with the control by the Student's t-test.

Differences in the distribution of phosphorylated proteins in trypomastigotes incubated with fibronectin or laminin were detected by immunofluorescence, as compared to parasites incubated with BSA, when a mixture containing anti-pS, -pT and -pY antibodies was employed (Fig. 1). The region of the flagellum attachment along the parasite body is devoid of labeling upon incubation with fibronectin whereas with laminin, a strong labeling at the posterior end is visualized (Fig. 1). Parasites with these modifications were quantitated and compared to the BSA control experiment. With fibronectin the visualized modifications were augmented 3-fold and with laminin 2.5-fold in relation to the control (Fig. 1B). These results suggest that distinct signaling pathways may be triggered upon adhesion of trypomastigotes to both fibronectin or laminin, although temporal differences in response to each of the ECM proteins cannot be ruled out.

**Figure 2:**
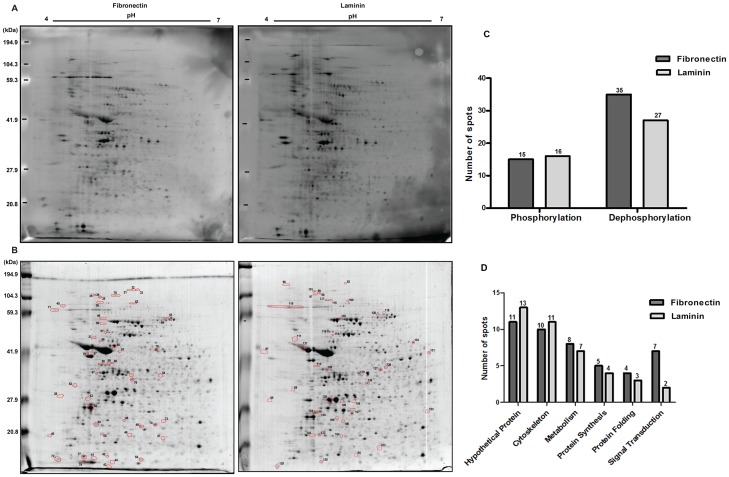
Trypomastigote proteins modified by phosphorylation/dephosphorylation upon adhesion to laminin or fibronectin. Incubation was for 2 h: (**A**) Phosphorylated proteins were stained with Pro-Q Diamond. (**B**) total protein profile developed with colloidal Coomassie blue staining; molecular mass markers (kDa) are shown on the ordinates; spots with variation in phosphorylation status are circled in red. (**C**) profile of phosphorylated and dephosphorylated spots upon 2 h incubation. (**D**) functional distribution of proteins from C.

To analyze the modifications at the protein level, trypomastigotes were added to tissue cultured plates previously coated with laminin, fibronectin or BSA. At different periods of time (5 min, 30 min, 1 h, 2 h, 3 h, 4 h), the parasites were collected and after washing, the whole-cell lysates were resolved by 2D-Electrophoresis and the phosphoproteins were detected by Pro-Q Diamond stain. The 2 h point was selected for further phosphoproteome analysis since no qualitative protein modifications were observed after this time (not shown). The phosphoproteome of fibronectin or laminin-incubated trypomastigotes (Fig. 2) showed 50 and 43 spots, respectively, differentially phosphorylated with statistical significance (ANOVA *p<0.05*), when compared to BSA-incubated trypomastigotes. These spots were then removed, subjected to trypsin digestion followed by LC-MS/MS analysis. Proteins were identified using Tritryp, the genome database of Kinetoplastids.

From the 50 phosphoproteome spots analyzed after incubation of trypomastigotes with fibronectin, 35 were dephosphorylated (total or partial reduction) and 15 had their phosphorylation levels increased (increase or phosphorylation emergency) (Table S1, Fig. 2C). Four spots submitted to analysis were not included in Table S1, since no match has been encountered. The majority of the sequenced spots corresponded to proteins with unknown function (11 spots, 23.9%) and cytoskeletal proteins (10 spots). In addition, proteins related to metabolic (8 spots) and signal transduction pathways (7 spots) were identified. Others were implicated in protein folding (4 spots) or in protein synthesis and degradation processes (5 spots) (Fig. 2D).

Upon trypomastigote treatment with laminin, from 43 spots analyzed, 27 showed reduction in the phosphorylation level (total or partial dephosphorylation), while 16 spots were phosphorylated (increment or phosphorylation emergency) (Table S2, Fig. 2C). Of these, 13 spots (34.2%) correspond to unknown proteins, 11 are components of the cytoskeleton, 7 belong to metabolic pathways, 4 are involved in protein synthesis and degradation processes, 3 in protein folding and 2 in signal transduction (Fig. 2D).

Considering both treatments, 20 sequenced spots with distinct phosphorylation status corresponded to proteins with no annotated function. Only two (access # Q4DCU5 and Q4E246) are common to fibronectin and laminin-treated parasites (Tables S1 and S2). Noticeably, the high numbers of proteins with unknown functions is a common finding in trypanosomatid proteomes up to now described [4]–[7],[23].

The majority of the identified spots showing phosphorylation changes after incubation of trypomastigotes with either fibronectin or laminin are related to cytoskeletal proteins (23.9%; 23.7%, respectively) (Fig. 2D), with higher incidence of hits to paraflagellar rod proteins and α- and β-tubulin. In addition, decrease in the phosphorylation level of heat shock proteins (HSP70, HSP60, HSP85) or at least of one of the proteasome subunits is common to both treatments. However, most of the proteins identified were differentially phosphorylated when both treatments were compared. EF2, for example, is dephosphorylated after incubation of trypomastigotes with fibronectin. This protein when phosphorylated in Thr^[169]^ inhibits translation initiation, a phenomenon related to the metacyclogenesis of *T. cruzi* [29]. Thus, attachment of the parasite to fibronectin may modulate the beginning of protein synthesis.

In addition to paraflagellar rod proteins and α- and β-tubulin, other proteins important for cytoskeleton and flagellar function were also modified at least in one of the experimental conditions, as for example flagellar radial spoke component, dyneins or I/6 protein. The latter, responsible for microtubules cross-linking [30], shows a significant phosphorylation in fibronectin-incubated trypomastigotes. However, the role of I/6 phosphorylation for microtubule function is unknown, as yet. Dephosphorylation of dynein light chain and radial spoke component occurs in trypomastigotes incubated with laminin or fibronectin, respectively. Dyneins, responsible for intracellular motility processes [31], are critical to flagella beating and viability of bloodstream forms of *T. brucei* [32]. The radial spoke, a component of the “9+2 flagella”, play an essential role in the activity of the dynein arms and in the flagellar wave, a cAMP- dependent phosphorylation regulatory process in *Chlamydomonas* [31],[33]. Moreover, other proteins implicated in signaling capacity belonging to the matrix or to the surface of the flagellum, as phosphoglycerate kinase and pyruvate kinase [23] were also identified. Both enzymes are underphosphorylated during adhesion of *T. cruzi* to laminin. Altogether, the data show the modification on the phosphorylation status of the cytoskeleton elements from *T. cruzi* in the adhesion to ECM elements.

Phosphorylation/dephosphorylation of cytoskeleton components in response to trypomastigotes adhesion to the extracellular matrix elements have been observed, implicating the involvement of tubulins and paraflagellar rod proteins in this phenomenon.

### Adhesion To Fibronectin Or Laminin-1 Induces α-Tubulin Dephosphorylation

Analyzing the 12 spots that match with α- or β- tubulin showing a variation in phosphorylation levels in response to incubation of trypomastigotes with fibronectin or laminin (Tables S1 and S2), it was found that all α- and β- tubulin spots analyzed have very significant score numbers, percentage of coverage and number of peptides sequenced, indicating a very successful protein identification. In general, adhesion of trypomastigotes to fibronectin leads to a strong dephosphorylation of α-tubulin (spots 37, 44, 64), and β-tubulin 1.9 (spot 58), as summarized in Table 1. It should be noted that isoform β-tubulin 1.9 (Q8STF3), which differs from β-tubulin (P08562) by 4 residues (S^274^, D^288^, L^289^ and A^306^ changed by the amino acids T, E, V, R in β-Tubulin 1.9, respectively), was described in other proteomic analysis of *T. cruzi* [4].

**Table 1:** Phosphorylation status of α-tubulin spots are modified after incubation of *T. cruzi* trypomastigotes with fibronectin or laminin-1*.

				Phosphorylation intensity		
Spot ID	Protein	Score - Mascot	Sequence Coverage (%)	Control	Fibronectin	ANOVA
37	alpha tubulin	74	3	12.19	0	7.13E-05
40	beta tubulin	69	7	0	3.19	1.02E-02
44	alpha tubulin	44	6	5.07	0	1.17E-03
58	beta tubulin 1.9	383	34	45.23	3.16	1.03E-02
64	alpha tubulin	201	13	8.07	0	2.61E-04
78	beta tubulin 1.9	296	18	1.79	2.74	3.39E-02
				Phosphorylation intensity		
Spot ID	Protein	Score - Mascot	Sequence Coverage (%)	Control	Laminin-1	ANOVA
**105**	alpha tubulin	120	9	1.99	2.37	3.39E-02
**106**	alpha tubulin	120	9	2.68	1.82	4.91E-02
**108**	alpha-tubulin	119	18	3.23	2.21	2.61E-02
**111**	alpha tubulin	78	4	7.43	0	3.40E-04
**113**	beta tubulin 1.9	383	34	12.90	1.98	3.25E-02
**137**	alpha tubulin	1252	39	1.93	2.78	3.36E-02

* Data were obtained from Tables S1 and S2.

doi:10.1371/journal.pone.0046767.t001

**Figure 3:**
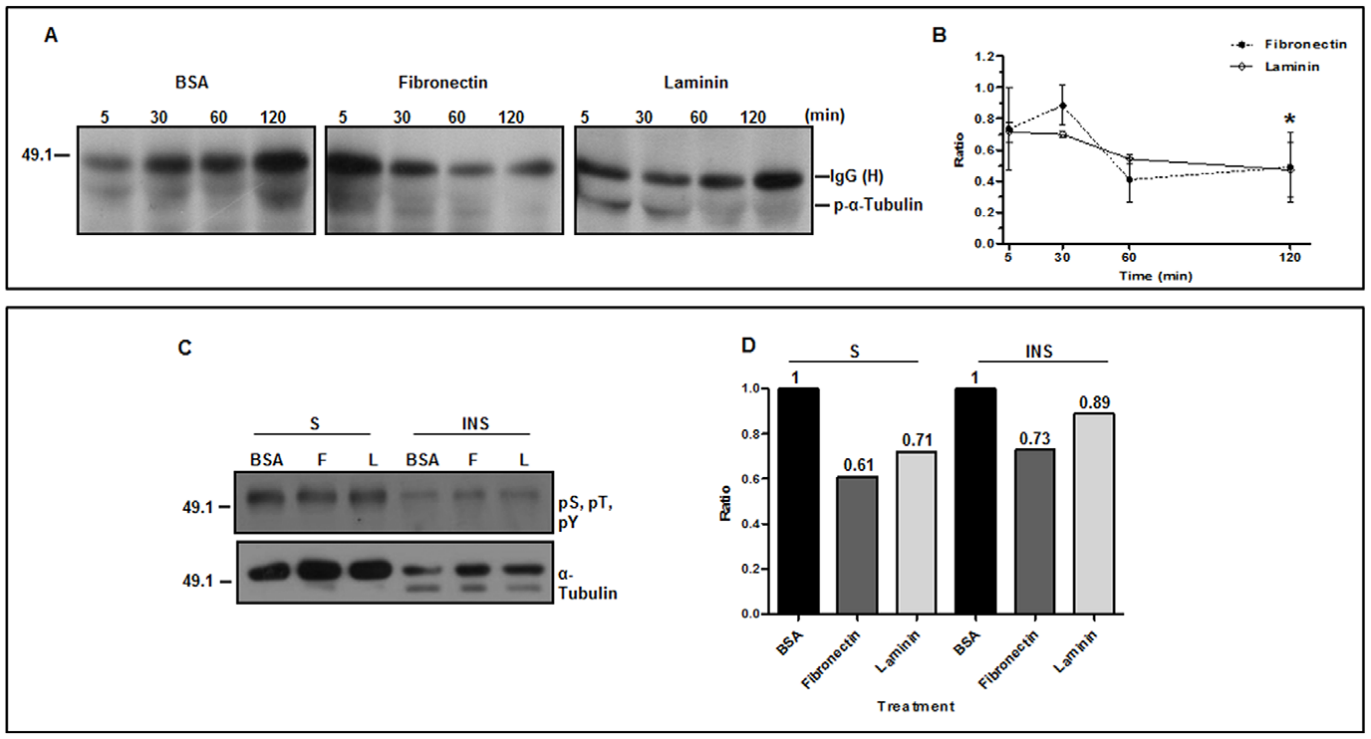
Phosphorylation status of α-tubulin upon adhesion of trypomastigotes to laminin, fibronectin or BSA. (**A**) Representative immunoblotting of immunoprecipitated α-tubulin phosphorylated (p-α-tubulin) during the times indicated. (**B**) Quantitation of 3 independent experiments as described in (**A**); asterisk represents a comparison between the 5 min and 120 min points by the Student's t-test with p<0.001, indicating a progressive α-tubulin dephosphorylation. (**C**) Representative immunoblotting of phosphorylated (pS, pT, pY, top) and total (bottom) soluble (S) and insoluble (INS) immunoprecipitated α-tubulin from trypomastigotes incubated for 2h with BSA, laminin (L) or fibronectin (F). (**D**) Calculation of the phosphorylation ratio relative to BSA from the experiments in (**C**); ratio: relative intensity of phosphorylated-tubulin treatment/control (BSA); on the left, 49.1 corresponds to the molecular mass standard in kDa.

Dephosphorylation of α-tubulin (spot 111) and β-tubulin 1.9 (spot 113) has also been observed upon incubation of trypomastigotes with laminin. Other spots, though, identified as α- or β-tubulins showed a slight increase in their phosphorylation levels when incubated with fibronectin (spots 40, 78) or laminin (spots 105, 137). A considerable number of α- and β- tubulin spots with low molecular mass were also identified (Table 1), a result also described for other trypanosomatids [34]-[36]. Since during experimental manipulation a cocktail of protease inhibitors was included, the lower molecular weight tubulins may have been generated by *in vivo* proteolysis. The predominance of α-tubulin dephosphorylation in the adhesion step of trypomastigotes to fibronectin or laminin was confirmed by immunoprecipitation. Extracts of trypomastigotes previously incubated with fibronectin, laminin or BSA for 5 min, 30 min, 60 min or 120 min were immunoprecipitated with α-tubulin-antibodies, followed by the development of the immunoblotting with a mixture of anti-pS, pT, pY antibodies. Dephosphorylation of α-tubulin is clearly observed after 1 h incubation of trypomastigotes with laminin, reaching approximately 65% after 2 h (Fig. 3A and B). Similar results were obtained after 2 h incubation of trypomastigotes with fibronectin. Dephosphorylation of α-tubulin could not be associated exclusively to a given particular tubulin fraction, since it was detected in the polymerized and in the soluble fraction of the cytoskeleton, although a slight increase in the soluble fraction could be observed (Fig. 3C and D). Thus, both, soluble and insoluble fractions of tubulin contribute to the total dephosphorylation detected in Fig. 3A and B.

In spite of the expressive dephosphorylation of α-tubulin, no changes in the morphological pattern of the cytoskeleton complex structure have been detected by immunofluorescence in parasites incubated for 2 h with laminin, fibronectin or BSA (control) (data not shown). This result strongly suggests that tubulin dephosphorylation is not correlated with microtubule remodeling.

### Paraflagellar-Rod Proteins Are Dephosphorylated In Fibronectin- And Laminin-Incubated Trypomastigotes

PAR proteins showed significant variation in phosphorylation levels after incubation of trypomastigotes with fibronectin or laminin. Good score values, percentage of coverage and number of peptides sequenced indicate a successful identification of PAR (Tables S1, S2, 2). Two of the 8 spots (spots 79 and 53) showed strong reduction in the phosphorylation status after treatment of trypomastigotes with fibronectin, while spot 49 showed a significant increase in phosphorylation (Table 2). In contrast, a less intense modification in the phosphorylation level of PAR (spots 118 and 135) was observed after incubation of trypomastigotes with laminin (Table 2). Interestingly, phosphorylation and dephosphorylation were observed predominantly in the lower molecular mass band of PAR (69 kDa). However, the existence of other less phosphorylated or less abundant isoforms cannot be excluded and may be due to lower reactivity of phosphorylated PAR with the antibodies.

**Table 2:** Phosphorylation status of Paraflagellar rod protein spots are modified after incubation of *T. cruzi* trypomastigotes with fibronectin or laminin-1*.

				Phosphorylation intensity		
Spot ID	Protein	Score - Mascot	Sequence Coverage (%)	Control	Fibronectin	ANOVA
**49**	major paraflagellar rod protein	164	28	2.44	10.04	1.26E-02
**53**	major paraflagellar rod protein	133	23	3.17	0	5.59E-03
**66**	major paraflagellar rod protein	435	35	1.80	3.11	3.86E-02
**79**	major paraflagellar rod protein	794	48	15.67	0	3.26E-05
				**Phosphorylation intensity**		
**Spot ID**	**Protein**	**Score - Mascot**	**Sequence Coverage (%)**	**Control**	**Laminin-1**	**ANOVA**
**118**	paraflagellar rod protein 3, putative	1076	68	1.80	2.48	4.29E-02
**120**	paraflagellar rod protein 3, putative	1076	68	11.55	2.38	2.62E-02
**135**	paraflagellar rod component	960	59	3.92	1.95	4.73E-02

* Data were obtained from Tables S1 and S2.

doi:10.1371/journal.pone.0046767.t002

**Figure 4:**
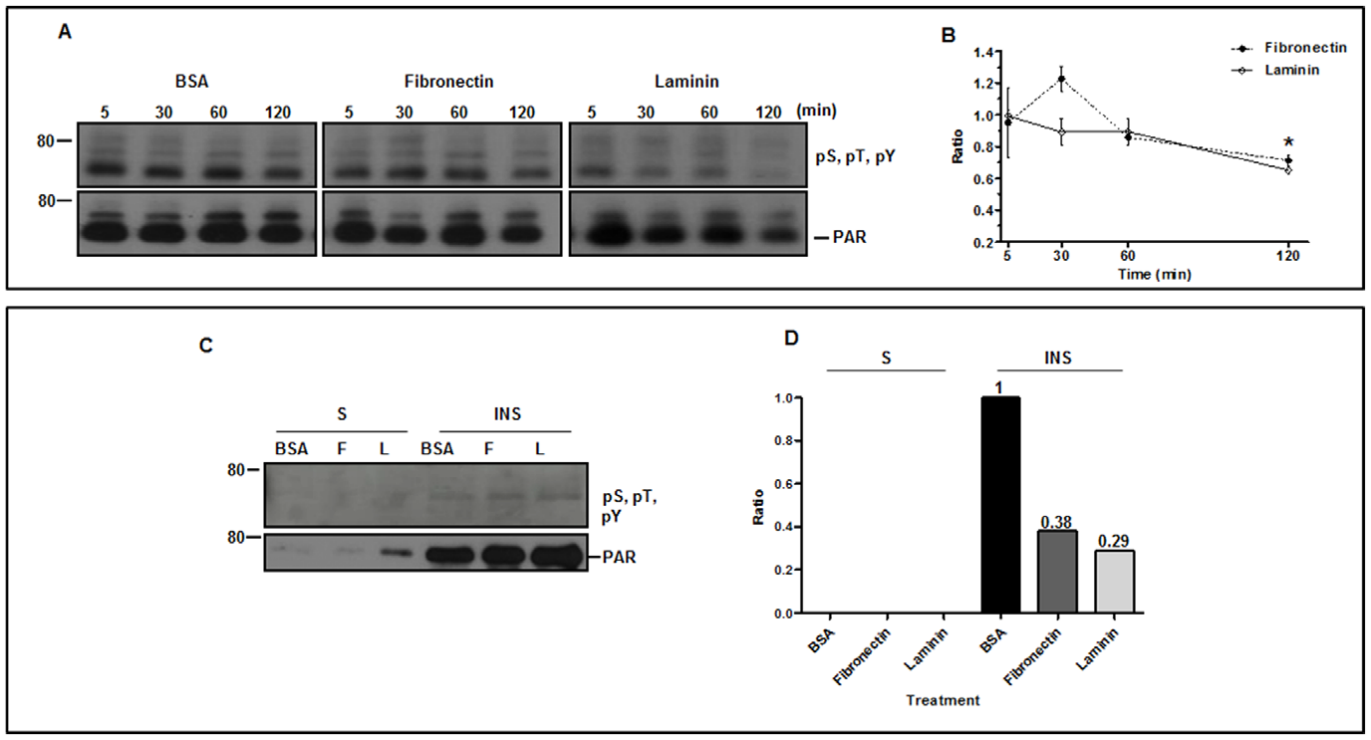
Phosphorylation status of PAR proteins upon incubation of trypomastigotes to laminin, fibronectin or BSA. (**A**) Representative immunoblotting of phosphorylated (pS, pT, pY) and PAR proteins immunoprecipitated during the incubation time. (**B**) Quantitation of 3 independent experiments as described in (**A**); asterisk represents a comparison between the 5 min and 120 min points by the Student's t-test with p<0.05, indicating a progressive PAR dephosphorylation. (**C**) Representative immunoblotting of phosphorylated (pS, pT, pY, top) and total (bottom) soluble (S) and insoluble (INS) immunoprecipitated PAR proteins from trypomastigotes incubated for 2 h with BSA, laminin (L) or fibronectin (F). (**D**) Calculation of the phosphorylation ratio relative to BSA from the experiments in (**C**); ratio: relative intensity of phosphorylated-PAR treatment/control (BSA); on the left, 80 corresponds to the molecular mass standard in kDa.

A major PAR dephosphorylation after incubation of the parasites with laminin or fibronectin was corroborated by immunoprecipitation of the extracts of trypomastigotes incubated with fibronectin, laminin or BSA for 5 min, 30 min, 60 min or 120 min, with anti-PAR MAb followed by immunoblottings using a mixture of anti-pS, pT, pY and anti-PAR MAb (Fig. 4A and B). Similarly to α-tubulin, a time dependent-reduction in the phosphorylation of PAR proteins can be observed, reaching approximately 40% and 30%, respectively, after 2 h incubation with laminin or fibronectin. All protein dephosphorylation was associated with the PAR structure, since it was detected only in the insoluble fraction of parasites treated with either laminin or fibronectin (Fig. 4C and 4D). Moreover, most of the PAR proteins recognized by the PAR MAb were detected in the insoluble fraction of the parasite extract (Fig. 4C and D).

To check whether reduction in PAR protein phosphorylation leads to any phenotypic change, p-formaldehyde fixed trypomastigotes previously incubated for 2 h with fibronectin, laminin or BSA (control) for 2 h were analyzed by immunofluorescence. No morphological differences could be detected in the distribution of PAR proteins by immunofluorescence microscopy upon adhesion, when compared to the control (data not shown). These findings together with those for α-tubulin strongly suggest that the parasite cytoskeleton dynamics is not affected by dephosphorylation events.

### The Erk Pathway Is Involved In The Response Of Trypomastigotes To Fibronectin Or Laminin-1 Adhesion

The mitogen activated kinase (MAPK) signal transduction pathway and, in particular ERK (extracellular signal-regulated kinase) signaling, play a fundamental role in diverse cellular functions and have been associated to fibronectin [37] and laminin-induced signaling receptors [38],[39]. The existence of ERK 1/2 pathway was also shown in *T. cruzi* [5],[40], *T. brucei* [6] and *Leishmania* [41]. Since signaling pathways in trypanosomatids are largely unknown, the possibility of ERK acting in *T. cruzi* trypomastigotes in response to fibronectin or laminin incubation in a time course experiment (5 min, 30 min, 60 min and 120 min) was then evaluated.

**Figure 5:**
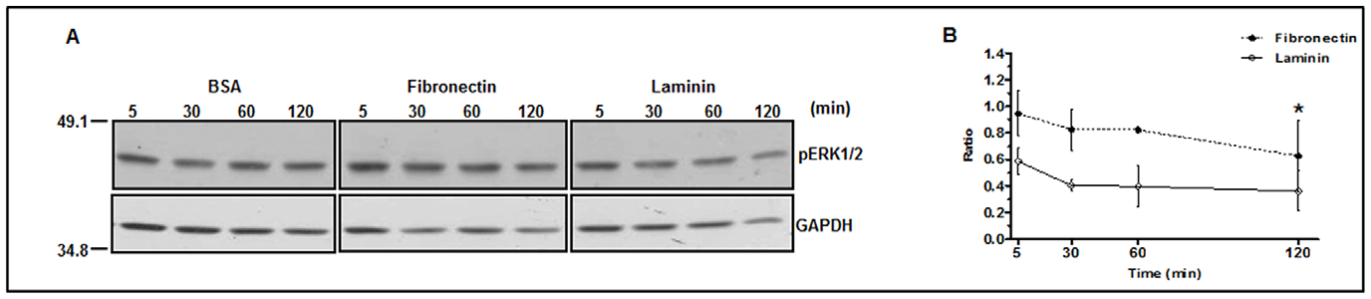
Phosphorylation status of ERK1/2 in trypomastigotes incubated with laminin, fibronectin or BSA. (**A**) Representative immunoblotting of phosphorylated ERK 1/2 (p-ERK 1/2) or GAPDH protein during the times indicated. (**B**) Calculation of the ERK 1/2 phosphorylation ratio for each experimental point relative to BSA from 2 experiments as in (**A**); on the left, 49.1 and 34.8 correspond to molecular mass standards in kDa. Asterisks represent a comparison between the 5 min and 120 min points by the Student's t-test with p<0.05 for fibronectin and p<0.2 for laminin.

Reduction of ERK phosphorylation is clearly shown in the 60 and 120 min time points of trypomastigotes incubation with fibronectin, in contrast to the incubation with BSA (control) (Fig. 5A and B). However, the kinetics and the intensity of dephosphorylation were not identical when fibronectin- or laminin- treated parasites were compared (Fig. 5B), probably due to the fact that ERK was already dephosphorylated by 40% in the 5 min point with laminin.

In short, dephosphorylation of tubulins, paraflagellar rod proteins and ERK is a common response to parasite adhesion to laminin or fibronectin. Notwithstanding, presently, there is no evidence to ascertain which of these events precedes the others.

## Discussion

Interaction of different pathogens with ECM components is well described. This interaction triggers a chain of events on the host cell mediated by membrane receptors. Less studied, though, are the signaling pathways unleashed on the parasite as a result of this interaction. Here it is shown for the first time that phosphorylation-dephosphorylation events are triggered in *T. cruzi* upon trypomastigotes adhesion to fibronectin or laminin. A clear evidence that adhesion to laminin or fibronectin leads to phosphorylation/dephosphorylation events on *T. cruzi* trypomastigotes was seen by immunofluorescence images of phosphoproteins (Fig. 1). Differences in fluorescence intensity or sub-cellular localization of phosphoproteins when fibronectin- or laminin-incubated trypomastigotes were compared, pointed out to distinct responses in each case. Both modifications were statistically significant and should be further explored.

A picture in which dephosphorylation predominates over phosphorylation became apparent upon parasite incubation with fibronectin and laminin. Seventy three per cent of all proteins analyzed were specific for each treatment, which may be explained by different receptor types or magnitude and duration of the stimulus. Only 27% of the identified proteins modified by phosphorylation/desphosphorylation were common to both treatments, the majority being tubulins, PAR protein 3 and heat shock proteins (Hsp85, Hsp70, Hsp60). As predictable, PAR proteins and tubulins were identified in the proteome of all stages of *T. cruzi* [35],[42],[43]. In fact, parasite cytoskeletal proteins are one of the main targets of the signaling process, although no changes in the architecture of the microfilaments or paraflagellar rod were evident upon parasite adhesion to these ECM proteins. This observation suggests that dephosphorylation may be important for the control of the affinities among proteins belonging to the signaling chain pathways.

Due to the methodological approach employed, less abundant proteins or small differences in phosphorylation might have been missed. Other proteins that may have been modified in response to laminin or fibronectin and were not noticed in this report should then be expected. For example, one protein not detected in the phosphoproteomic analysis was ERK 1/2, known to be modified in response to ECM proteins in mammalian cells. In fact, using specific antibody to phosphorylated ERK1/2, dephosphorylation (inactivation) of that protein was observed. Interestingly, dephosphorylation of ERK was also observed in lung cancer cells after incubation with laminin [38]. On the other hand, phosphorylation of ERK (activation) leading to proliferation or motility is a normal response when cells adhere to fibronectin through integrin interactions [37]. It should be mentioned that the corresponding ERK 2 in *T. cruzi* (TcMAPK2) is present in all stages of the parasite, although in a more phosphorylated (active) form in trypomastigotes in which is localized along the flagellar contour [40]. The results indicate that ERK dephosphorylation is part of the *T. cruzi* response to adhesion to laminin and fibronectin suggesting that the downstream cascade should be further exploited.

The phosphorylation sites of tubulin were not identified in *T. cruzi*. It should be noted that ser/thr kinase CK2 was described to be tightly associated with the parasite heterodimeric α/β tubulins and capable of phosphorylating α and β tubulins *in vitro* [44],[45]. Moreover, phosphorylation of serine was identified in *T. brucei* β-tubulin (GLSVPELTQQMFDAK) [6]. The precise function of the tubulin phosphorylation control is under discussion [46]–[49]. Mutation on Ser^172^ β-tubulin leads to defects in microtubule dynamics and cell division in yeast [50], phosphorylation by Cdk1 kinase inhibits tubulin dimer addition to microtubules *in vitro* [51] and phosphorylation on Ser^165^ α6 tubulin by PKCα leads to microtubule elongation and motility in human breast cancer cells [52]. Moreover, phosphorylation of α- tubulin on tyrosine residues was found only in the unpolymerized soluble fraction and was implicated in polymerization of microtubules in T lymphocytes [53].

Much less is known about the role of phosphatases in microtubular function. For example, target mutagenesis of the Ppp1cc gene results in severe defect in spermatogenesis, with high phosphorylation of α- and β-tubulin isoforms, leading to the disorganization of the tubulin network [54]. Moreover, βIII-tubulin isoform and Tau (axonal microtubule associated protein) are preferentially dephosphorylated by protein phosphatase 2A, in neuron microtubules [55]. In *T. cruzi*, a strong association of protein phosphatase 2A (PP2A) with α-tubulin, myosin and actin has been reported, suggesting a role in trypomastigote transformation into amastigotes and flagellum shortening [56]. Thus, the possibility that dephosphorylation of tubulin may affect parasite motility or the affinity of parasite ligands to host receptor molecules should be further investigated.

As happens with tubulins, the role of the phosphorylation/dephosphorylation cycle of paraflagellar rod proteins is unknown. PAR, a structure restricted to kinetoplastids, euglenoids and dinoflagellates, is composed mainly by PFR1 (PAR3) and PFR2 (PAR2) [57],[58]. Phosphorylation of one PAR protein in a CAMK2 consensus sequence (RxxS/T) was described in the proteome of *T. cruzi* epimastigotes [4],[5] and sequences containing phosphorylated serine and threonine were described in *T. brucei* [6]. In addition to the well established role in flagellar motility [59],[60], PAR may integrate and transmit to the cell body or to the axoneme, the external signals captured by the flagellum [20]. The modifications of the phosphorylation status of flagellar protein herein described (Tables S1, S2, 2 and Fig. 4) may be involved in a hypothetical signal mediation by the flagellum when interacting with host cell ECM. This hypothesis is reinforced by the dephosphorylation of ERK 1/2 described in the process (Fig. 5). It is worth noticing the presence of ERK 1/2 in the flagellum [40] together with other kinases and phosphatases.

Many issues have to be addressed to understand completely the signal transduction upon adhesion of trypomastigotes to ECM. The role of phosphatases and kinases should be investigated as well as the modulation of the phosphorylation status of α-tubulin and PAR proteins as scaffold structures in the signaling process. ERK dephosphorylation and the ensuing effect in downstream elements should be more investigated. Since it is well established that trypomastigotes interact with ECM elements through members of the gp85 GPI-anchored surface glycoprotein family, as Tc85 or gp83 [61], it is tempting to hypothesize that adhesion of gp85 proteins to laminin or fibronectin may trigger signal transduction.

The knowledge on the modifications of microtubule and paraflagellar rod protein phosphorylation relative to adhesion of *T. cruzi* trypomastigotes to ECM elements, herein described for the first time, is at its inception. Phosphatases activation, cytoskeleton dephosphorylation, and ERK 1/2 inactivation may form a chain of events leading to the success of parasite invasion. In this context, the cytoskeletal proteins may directly participate in the signaling pathway that is activated during parasite attachment to the host cell.

This novel hypothesis will be the focus of future investigations aiming at getting clues into the mechanism employed by *T. cruzi* to invade cells.

## Supporting Information

Table S1 Identification of differentially phosphorylated proteins after incubation of *T. cruzi* trypomastigotes with fibronectin. (DOC)

Table S2 Identification of differentially phosphorylated proteins after incubation of *T. cruzi* trypomastigotes with laminin-1. (DOC)
