# Supplementary material for: Adhesion of Trypanosoma cruzi Trypomastigotes to Fibronectin or Laminin Modifies Tubulin and Paraflagellar Rod Protein Phosphorylation
Source: PLoS One. 2012 Oct 4;7(10):e46767. doi: 10.1371/journal.pone.0046767 (PMC3465109; doi:10.1371/journal.pone.0046767)
Supplement: Table S1 — (DOC) [file pone.0046767.s001.doc]

|  |  |  |  |  |  | **Theoretical** | | **Experimental** | | **Phosphorylation Intensity** | |  |
| --- | --- | --- | --- | --- | --- | --- | --- | --- | --- | --- | --- | --- |
| **Spot ID** | **Protein** | **Uniprot Entry #** | **Score - Mascot** | **No. of Peptides Identified** | **Sequence Coverage (%)** | **pI** | **MW (Da)** | **pI** | **MW (Da)** | **Control** | **Fibronectin** | **ANOVA** |
| **33** | GPI anchor biosynthesis protein, putative | Q4CZK3 | 29 | 1 | 1 | 9.41 | 67,768 | 5.87 | 123,67 | 3.61 | 0 | 3.62E-03 |
| **35** | hypothetical protein, conserved | Q4DCU5 | 99 | 8 | 8 | 4.89 | 120,565 | 6.41 | 89,29 | 8.04 | 0 | 2.64E-04 |
| **36** | lipophosphoglycan biosynthetic protein, putative | Q4DW89 | 39 | 5 | 4 | 5.26 | 86,969 | 5.33 | 104,329 | 6.20 | 3.27 | 8.73E-03 |
| **37** | alpha tubulin | Q27352 | 74 | 1 | 3 | 4.9 | 50,324 | 5.43 | 110,065 | 12.19 | 0 | 7.13E-05 |
| **39** | hypothetical protein, conserved | Q4D1U0 | 314 | 25 | 21 | 5.02 | 152,658 | 4.61 | 33,899 | 2.40 | 3.19 | 1.80E-02 |
| **40** | beta tubulin | P08562 | 69 | 3 | 7 | 4.69 | 50,225 | 5.26 | 113,69 | 0 | 3.19 | 1.02E-02 |
| **41** | I/6 autoantigen, putative | Q4DFL2 | 278 | 8 | 41 | 5.19 | 23,439 | 4.46 | 20,192 | 0 | 5.23 | 1.84E-03 |
| **42** | hypothetical protein, conserved | Q4D0B5 | 52 | 3 | 11 | 5.23 | 28,401 | 5.19 | 15,564 | 124.12 | 0 | 6.04E-08 |
| **43** | hypothetical protein, conserved | Q4CNF7 | 75 | 2 | 9 | 5.5 | 26,379 | 5.38 | 17,318 | 0 | 4.21 | 3.85E-03 |
| **44** | alpha tubulin | Q27352 | 44 | 3 | 6 | 4.9 | 50,324 | 5.48 | 9,911 | 5.07 | 0 | 1.17E-03 |
| **45** | hypothetical protein, conserved | Q4E246 | 110 | 11 | 11 | 4.64 | 102,819 | 5.53 | 10,328 | 8.50 | 2.04 | 4.10E-02 |
| **46** | hslvu complex proteolytic subunit-like, putative | Q4D729 | 105 | 5 | 23 | 6.77 | 23,038 | 4.75 | 98,558 | 3.23 | 0 | 5.23E-03 |
| **47** | leucine-rich repeat protein, putative | Q4CLM9 | 48 | 9 | 35 | 5.82 | 26,926 | 5.78 | 20,734 | 6.09 | 0 | 6.42E-04 |
| **48** | adenylate kinase, putative | Q4D6Z4 | 607 | 20 | 74 | 5.49 | 29,437 | 5.79 | 19,607 | 2.77 | 3.26 | 1.26E-02 |
| **49** | major paraflagellar rod protein | Q01530 | 164 | 19 | 28 | 5.85 | 69,961 | 6.25 | 25,085 | 2.44 | 10.04 | 1.26E-02 |
| **51** | heat shock protein 70 | Q56UI2 | 125 | 13 | 21 | 5.06 | 71,444 | 5.47 | 86,248 | 5.95 | 3.98 | 5.06E-03 |
| **52** | heat shock protein, putative | Q4DI67 | 156 | 12 | 15 | 5.28 | 94,038 | 5.38 | 68,097 | 1.87 | 2.22 | 3.87E-02 |
| **53** | major paraflagellar rod protein | Q01530 | 133 | 14 | 23 | 5.85 | 69,961 | 5.82 | 101,197 | 3.17 | 0 | 5.59E-03 |
| **54** | asparagine synthetase A, putative | Q4CRM4 | 83 | 8 | 20 | 5.83 | 39,305 | 6.35 | 85,685 | 2.77 | 3.52 | 1.16E-02 |
| **55** | hypothetical protein, conserved | Q4CVX7 | 111 | 8 | 20 | 5.28 | 41,12 | 6.38 | 48,335 | 6.09 | 0 | 6.43E-04 |
| **56** | heat shock protein 60 kDa | Q95046 | 168 | 9 | 18 | 5.38 | 59,602 | 5.80 | 45,036 | 4.48 | 0 | 1.75E-03 |
| **57** | Hsc70-interacting protein (Hip), putative | Q4DAT6 | 175 | 9 | 17 | 4.83 | 48,233 | 5.38 | 77,674 | 1.74 | 2.01 | 3.98E-02 |
| **58** | beta tubulin 1.9 | Q8STF3 | 383 | 17 | 34 | 4.74 | 50,352 | 5.20 | 56,552 | 45.23 | 3.16 | 1.03E-02 |
| **59** | proteasome regulatory ATPase subunit 2, putative | Q4D0B9 | 61 | 1 | 2 | 5.51 | 49,245 | 5.50 | 52,144 | 3.22 | 0 | 5.29E-03 |
| **60** | heat shock protein 85, putative | Q4CQS6 | 164 | 12 | 19 | 5.07 | 81,132 | 6.00 | 60,729 | 2.15 | 0 | 2.14E-02 |
| **61** | vacuolar ATP synthase subunit B, putative | Q4CXR7 | 30 | 2 | 4 | 5.43 | 55,847 | 5.59 | 48,028 | 20.85 | 8.08 | 4.88E-04 |
| **62** | nucleoside hydrolase, putative | Q4DUW5 | 179 | 14 | 41 | 4.66 | 40,052 | 5.62 | 57,348 | 0 | 3.53 | 7.21E-03 |
| **63** | hypothetical protein, conserved | Q4DX47 | 147 | 8 | 28 | 4.86 | 29,114 | 4.91 | 38,46 | 2.47 | 1.94 | 4.41E-02 |
| **64** | alpha tubulin | Q27352 | 201 | 9 | 13 | 4.9 | 50,324 | 5.08 | 32,57 | 8.07 | 0 | 2.61E-04 |
| **65** | proteasome beta 3 subunit, putative | Q4DHA9 | 36 | 1 | 4 | 5 | 22,819 | 5.98 | 10,748 | 3.94 | 0 | 2.70E-03 |
| **66** | major paraflagellar rod protein | Q01530 | 435 | 28 | 35 | 5.85 | 69,961 | 5.34 | 16,021 | 1.80 | 3.11 | 3.86E-02 |
| **67** | hypothetical protein, conserved | Q4DBH2 | 84 | 5 | 14 | 4.83 | 38,831 | 6.33 | 84,712 | 2.04 | 0 | 2.58E-02 |
| **68** | hypothetical protein, conserved | Q4D5L9 | 63 | 2 | 7 | 5.21 | 29,124 | 5.34 | 41,282 | 8.36 | 0 | 2.33E-04 |
| **69** | 14-3-3 protein | Q6B9P3 | 358 | 15 | 45 | 4.99 | 30,06 | 5.75 | 20,487 | 1.96 | 0 | 3.14E-02 |
| **70** | hypothetical protein, conserved | Q4DZS2 | 319 | 21 | 22 | 5.17 | 102,109 | 5.32 | 18,262 | 2.53 | 1.88 | 4.89E-02 |
| **71** | flagellar radial spoke component, putative | Q4DE57 | 195 | 13 | 22 | 4.3 | 67,897 | 5.59 | 112,818 | 2.93 | 2.01 | 3.34E-02 |
| **72** | hypothetical protein, conserved | Q4DJJ4 | 235 | 9 | 34 | 4.3 | 19,572 | 4.63 | 93,593 | 0 | 2.70 | 1.82E-02 |
| **73** | elongation factor 2 | Q6IWF6 | 105 | 5 | 7 | 5.79 | 95,084 | 4.67 | 10,748 | 2.01 | 0 | 2.70E-02 |
| **74** | thiol transferase Tc52 | A2TEK0 | 124 | 7 | 11 | 5.74 | 51,017 | 6.44 | 26,806 | 3.20 | 2.58 | 1.61E-02 |
| **75** | Hsc70-interacting protein (Hip), putative | Q4DAT6 | 415 | 18 | 35 | 4.83 | 48,233 | 6.31 | 23,567 | 3.09 | 2.80 | 1.47E-02 |
| **76** | asparagine synthetase A, putative | Q4CRM4 | 303 | 17 | 49 | 5.83 | 39,305 | 5.27 | 51,981 | 3.37 | 0 | 3.48E-04 |
| **77** | eukaryotic initiation factor 5a, putative | Q4E4N4 | 186 | 7 | 55 | 4.82 | 18,118 | 5.84 | 42,532 | 0 | 2.98 | 1.09E-02 |
| **78** | beta tubulin 1.9 | Q8STF3 | 296 | 10 | 18 | 4.74 | 50,352 | 4.99 | 11,171 | 1.79 | 2.74 | 3.39E-02 |
| **79** | major paraflagellar rod protein | Q01530 | 794 | 41 | 48 | 5.85 | 69,961 | 5.00 | 9,911 | 15.67 | 0 | 3.26E-05 |
| **80** | ATPase beta subunit, putative | Q4DTX7 | 1205 | 29 | 64 | 5.27 | 55,924 | 6.24 | 59,52 | 2.21 | 3.40 | 2.14E-02 |
| **81** | activated protein kinase C receptor, putative | Q4DTN2 | 900 | 29 | 77 | 5.73 | 35,522 | 5.59 | 59,983 | 2.77 | 3.52 | 1.16E-02 |
