# Supplementary material for: Adhesion of Trypanosoma cruzi Trypomastigotes to Fibronectin or Laminin Modifies Tubulin and Paraflagellar Rod Protein Phosphorylation
Source: PLoS One. 2012 Oct 4;7(10):e46767. doi: 10.1371/journal.pone.0046767 (PMC3465109; doi:10.1371/journal.pone.0046767)
Supplement: Table S2 — (DOC) [file pone.0046767.s002.doc]

|  |  |  |  |  |  | **Theoretical** | | **Experimental** | | **Phosphorylation Intensity** | |  |
| --- | --- | --- | --- | --- | --- | --- | --- | --- | --- | --- | --- | --- |
| **Spot ID** | **Protein** | **Uniprot Entry #** | **Score - Mascot** | **No. of Peptides Identified** | **Sequence Coverage (%)** | **pI** | **MW (Da)** | **pI** | **MW (Da)** | **Control** | **Laminin-1** | **ANOVA** |
| **83** | dynein light chain, putative | Q4CZW3 | 23 | 1 | 3 | 6.61 | 29,584 | 5.83 | 122,394 | 6.12 | 0 | 6.34E-04 |
| **84** | proteasome alpha 2 subunit, putative | Q4DAW6 | 45 | 2 | 11 | 5.74 | 25,266 | 6.01 | 9,952 | 2.83 | 2.66 | 2.08E-01 |
| **85** | hypothetical protein, conserved | Q4D4S4 | 31 | 3 | 10 | 4.61 | 27,136 | 4.59 | 29,195 | 7.91 | 3.32 | 6.37E-03 |
| **87** | hypothetical protein, conserved | Q4CPU7 | 64 | 1 | 3 | 4.33 | 38,005 | 4.52 | 53,217 | 2.23 | 1.90 | 3.21E-02 |
| **88** | nucleoside hydrolase, putative | Q4DUW5 | 177 | 14 | 41 | 4.66 | 40,052 | 6.36 | 33,777 | 0 | 2.23 | 2.69E-02 |
| **96** | hypothetical protein, conserved | Q4E246 | 200 | 16 | 17 | 4.64 | 102,819 | 4.88 | 119,436 | 0 | 1.98 | 4.84E-02 |
| **97** | hypothetical protein, conserved | Q4DCU5 | 39 | 5 | 5 | 4.89 | 120,565 | 5.34 | 104,329 | 8.10 | 0 | 2.57E-04 |
| **99** | hypothetical protein, conserved | Q4CZW1 | 29 | 1 | 2 | 8.99 | 39,756 | 5.42 | 105,666 | 9.74 | 0 | 1.44E-04 |
| **100** | hypothetical protein, conserved | Q4DRF1 | 102 | 9 | 11 | 5.44 | 90,788 | 5.38 | 106,835 | 1.83 | 0 | 3.77E-02 |
| **101** | hypothetical protein, conserved | Q4DCU5 | 908 | 40 | 41 | 4.89 | 120,565 | 5.87 | 96,026 | 9.51 | 0 | 1.55E-04 |
| **103** | proteasome beta 7 subunit, putative | Q4D579 | 29 | 3 | 7 | 5.61 | 24,766 | 6.39 | 11,982 | 0 | 2.39 | 2.41E-02 |
| **104** | heat shock protein HSP70 | Q26936 | 89 | 7 | 11 | 5.42 | 71,495 | 5.77 | 15,975 | 25.50 | 7.59 | 5.72E-04 |
| **105** | alpha tubulin | Q27352 | 120 | 4 | 9 | 4.9 | 50,324 | 5.70 | 22,438 | 1.99 | 2.37 | 3.39E-02 |
| **106** | alpha tubulin | Q27352 | 120 | 4 | 9 | 4.9 | 50,324 | 5.80 | 20,932 | 2.68 | 1.82 | 4.91E-02 |
| **107** | dihydrolipoamide acetyltransferase, putative | Q4DYI5 | 287 | 8 | 31 | 6.62 | 28,304 | 5.95 | 25,458 | 1.75 | 2.36 | 4.68E-02 |
| **108** | alpha-tubulin | Q26973 | 119 | 8 | 18 | 5.49 | 47,498 | 5.97 | 28,856 | 3.23 | 2.21 | 2.61E-02 |
| **109** | hypothetical protein, conserved | Q4DRY8 | 59 | 3 | 8 | 5.25 | 37,324 | 5.77 | 28,856 | 6.56 | 3.33 | 8.27E-03 |
| **110** | hypothetical protein Tc00.1047053504013.20 | Q4D6P7 | 36 | 1 | 2 | 6.4 | 28,805 | 6.09 | 40,871 | 3.04 | 1.94 | 4.72E-02 |
| **111** | alpha tubulin | Q27352 | 78 | 2 | 4 | 4.9 | 50,324 | 5.02 | 61,2 | 7.43 | 0 | 3.40E-04 |
| **113** | beta tubulin 1.9 | Q8STF3 | 383 | 17 | 34 | 4.74 | 50,352 | 5.37 | 45,11 | 12.90 | 1.98 | 3.25E-02 |
| **114** | heat shock protein 60 kDa | Q95046 | 87 | 2 | 4 | 5.38 | 59,602 | 5.63 | 64,503 | 70.29 | 0 | 3.36E-07 |
| **115** | chaperonin containing T-complex protein, putative | Q4DWG6 | 63 | 5 | 8 | 5.05 | 60,004 | 5.55 | 66,635 | 2.38 | 0 | 1.51E-02 |
| **116** | hypothetical protein, conserved | Q4DCU5 | 164 | 12 | 11 | 4.89 | 120,565 | 5.59 | 97,363 | 0 | 3.49 | 7.43E-03 |
| **117** | hypothetical protein, conserved | Q4DCU5 | 105 | 9 | 8 | 4.89 | 120,565 | 5.52 | 96,858 | 5.21 | 0 | 1.07E-03 |
| **118** | paraflagellar rod protein 3, putative | Q4D634 | 1076 | 53 | 68 | 5.81 | 69,076 | 6.46 | 84,162 | 1.80 | 2.48 | 4.29E-02 |
| **119** | heat shock protein 85, putative | Q4CQS6 | 907 | 39 | 41 | 5.07 | 81,132 | 5.60 | 85,67 | 3.62 | 2.38 | 1.72E-02 |
| **120** | paraflagellar rod protein 3, putative | Q4D634 | 1076 | 53 | 68 | 5.81 | 69,076 | 6.44 | 82,627 | 11.55 | 2.38 | 2.62E-02 |
| **122** | 14-3-3 protein | Q6B9P3 | 68 | 3 | 13 | 4.99 | 30,06 | 5.30 | 16,158 | 1.94 | 0 | 3.08E-02 |
| **123** | 14-3-3 protein | Q6B9P3 | 146 | 12 | 37 | 4.99 | 30,06 | 5.42 | 16,481 | 4.33 | 19.69 | 1.96E-03 |
| **125** | hypothetical protein, conserved | Q4CS20 | 206 | 10 | 48 | 5.01 | 23,12 | 5.44 | 7,906 | 5.21 | 0 | 1.07E-03 |
| **126** | glycyl-tRNA synthetase, putative | Q4D0Z5 | 46 | 2 | 6 | 5.96 | 37,314 | 6.49 | 44,455 | 4.38 | 3.65 | 5.71E-03 |
| **127** | pyruvate kinase 2, putative | Q4E1U3 | 562 | 19 | 30 | 7.11 | 55,38 | 6.40 | 43,379 | 2.12 | 0 | 2.25E-02 |
| **128** | ATPase beta subunit, putative | Q4DTX7 | 209 | 7 | 14 | 5.27 | 55,924 | 5.53 | 43,522 | 23.53 | 2.24 | 2.75E-02 |
| **129** | phosphoglycerate kinase, putative | Q4D193 | 412 | 21 | 52 | 6.19 | 44,783 | 4.72 | 6,592 | 0 | 11.48 | 1.53E-04 |
| **130** | aminopeptidase, putative | Q4DZJ3 | 452 | 20 | 41 | 6.06 | 56,769 | 5.42 | 24,663 | 3.83 | 0 | 2.96E-03 |
| **131** | hypothetical protein, conserved | Q4E1W3 | 178 | 11 | 32 | 5.6 | 37,492 | 7.20 | 52,637 | 8.05 | 2.18 | 3.49E-02 |
| **132** | phosphoglycerate kinase, putative | Q4D193 | 412 | 18 | 53 | 6.19 | 44,783 | 6.94 | 59,336 | 0 | 2.26 | 3.39E-02 |
| **133** | ribosomal protein P0 | Q4E3A4 | 340 | 18 | 34 | 4.91 | 35,221 | 7.17 | 24,663 | 1.73 | 2.31 | 3.65E-02 |
| **135** | paraflagellar rod component | O00930 | 960 | 46 | 59 | 5.19 | 69,144 | 5.76 | 77,552 | 3.92 | 1.95 | 4.73E-02 |
| **137** | alpha tubulin | Q8T9X5 | 1252 | 36 | 39 | 4.94 | 50,395 | 5.14 | 55,854 | 1.93 | 2.78 | 3.36E-02 |
